# Incidence of Guillain–Barré Syndrome Following COVID-19 Vaccination and SARS-CoV-2 Infection: A Population-Based Cohort Study Using the Valencia Health System Integrated Database (Spain)

**DOI:** 10.3390/ph19030477

**Published:** 2026-03-14

**Authors:** Elisa Correcher-Martínez, Sergio Pascual Viciedo-Mata, Arantxa Urchueguía-Fornes, Juan José Carreras

**Affiliations:** 1 Vaccine Research Department, Foundation for the Promotion of Health and Biomedical Research in the Valencian Region (FISABIO—Public Health), 46020 Valencia, Spain; elisa.correcher@fisabio.es (E.C.-M.); arantxa.urchueguia@fisabio.es (A.U.-F.); 2 Subdirectorate of Epidemiology and Public Health Surveillance, Autonomous Community of Valencia, 46020 Valencia, Spain; viciedo_ser@gva.es; 3 Biomedical Research Consortium of Epidemiology and Public Health (CIBER-ESP), Instituto de Salud Carlos III, 28029 Madrid, Spain

**Keywords:** Guillain–Barré Syndrome, COVID-19 vaccines, SARS-CoV-2 infection, vaccine safety, pharmacoepidemiology, pharmacovigilance, real-world data

## Abstract

**Background/Objectives**: Guillain–Barré syndrome (GBS) is a rare but serious immune-mediated neurological disorder monitored as an adverse event of special interest during the COVID-19 pandemic. This study aimed to estimate the incidence of GBS following COVID-19 vaccination and SARS-CoV-2 infection and to compare the risk by vaccine platform. **Methods**: We conducted a population-based retrospective cohort study using data from the Valencia Health System Integrated Database (Spain) between January 2018 and March 2022. Two cohorts were defined: individuals receiving COVID-19 vaccines (mRNA-based vaccines (BNT162b2 [Pfizer–BioNTech] and mRNA-1273 [Moderna]) or non-virus-vectored (NVV) adenoviral vector-based vaccines (ChAdOx1-S [AstraZeneca] and Ad26.COV2.S [Janssen])) and individuals with laboratory-confirmed SARS-CoV-2 infection. Incident GBS cases were identified within a predefined 42-day risk window following vaccination or infection. Incidence rates were calculated per 100,000 vaccine doses administered or SARS-CoV-2 infections. **Results**: Among 5,109,919 individuals, 4,270,610 received at least one COVID-19 vaccine dose and 920,643 experienced at least one SARS-CoV-2 infection. A total of 69 GBS cases occurred within 42 days following vaccination (incidence: 0.67 per 100,000 doses), whereas 21 cases occurred after infection (incidence: 2.20 per 100,000 infections). Incidence was lower after mRNA-based vaccines (0.55 per 100,000 doses) than after NVV vaccines (1.57 per 100,000 doses). **Conclusions**: This study confirms that GBS occurrence following vaccination is rare. The incidence is lower among individuals who received mRNA vaccines compared to those who received NVV vaccines. Moreover, GBS appears to be more frequent after a COVID-19 infection than after vaccination. These findings highlight the importance of integrating pharmacoepidemiological analyses with pharmacovigilance data to contextualize rare but serious adverse events.

## 1. Introduction

Guillain–Barré Syndrome (GBS) is a rare but potentially life-threatening autoimmune disorder in which the immune system targets the peripheral nervous system, leading to progressive weakness, sensory disturbances, and, in severe cases, respiratory failure [1]. Globally, GBS is the leading cause of acute flaccid paralysis, surpassing poliomyelitis, which has been nearly eradicated thanks to global vaccination efforts [2,3]. While GBS can affect individuals of all ages, it is more prevalent in males and older adults, with incidence rates increasing with age. In developed countries, the annual incidence is estimated to be 1–2 cases per 100,000 individuals, and approximately 5% of patients succumb to complications such as respiratory failure, cardiovascular instability, or infections [1,2,4]. Although treatments like intravenous immunoglobulin and plasma exchange have improved outcomes for GBS patients, a significant proportion still suffer from long-term disabilities. These disabilities often require extended hospitalization and rehabilitation, with many individuals requiring assistance to regain motor functions [2,5].

The etiology of GBS is not yet fully understood. However, it is widely accepted that the condition is often triggered by an aberrant immune response following a viral or bacterial infection, particularly those affecting the respiratory or gastrointestinal systems [6,7]. Research suggests that up to 60% of GBS cases are preceded by infections such as *Campylobacter jejuni*, a common cause of bacterial gastroenteritis, as well as viruses like Cytomegalovirus and Epstein–Barr virus [8]. Additionally, GBS has been associated with various emerging infectious diseases, including Zika virus outbreaks, where increased incidences of GBS were reported in areas with high Zika infection rates [9,10].

Historically, concerns regarding a potential association between GBS and vaccination emerged following the 1976 influenza vaccination campaign, prompting the systematic monitoring of GBS in vaccine safety surveillance systems [11]. While the increased risk of GBS following influenza vaccination has been documented in some studies, the overall risk remains extremely low, and the benefits of vaccination outweigh the risks in most cases [12]. Given this historical precedent, GBS was identified as a potential Adverse Event of Special Interest (AESI) for surveillance systems monitoring Adverse Events Following Immunization (AEFI) during the global rollout of COVID-19 vaccines [13].

Accumulating evidence suggests that SARS-CoV-2 infection itself may act as a trigger for GBS, although the condition remains a rare complication of COVID-19 [14,15,16]. The exact pathogenic mechanisms through which the virus triggers GBS are still under investigation, but molecular mimicry and immune dysregulation are thought to play a role [17]. Additionally, a temporal association between GBS and non-virus-vectored (NVV) vaccines, corresponding in this study to adenoviral vector-based COVID-19 vaccines (ChAdOx1-S [AstraZeneca] and Ad26.COV2.S [Janssen]) has been reported, with GBS subsequently being listed as a potential rare side effect in these vaccines’ product information [18,19]. In contrast, evidence supporting an association between mRNA-based COVID-19 vaccines and GBS has remained limited and inconsistent [20]. The possibility of confounding factors and biases in the detection and reporting of GBS cases following vaccinations warrants further investigation.

From a methodological perspective, evaluating rare adverse events such as GBS poses important challenges. Spontaneous reporting systems are essential for signal detection but are prone to underreporting and reporting biases, whereas population-based healthcare databases enable active surveillance and risk quantification but may lack detailed clinical validation. Integrating pharmacoepidemiological analyses with pharmacovigilance data is therefore critical to contextualize risks, assess underreporting, and support regulatory decision-making.

In this context, the present study uses the Integrated Database of the Valencian Health System (VID) [21] to estimate the incidence of GBS within a 42-day risk window following COVID-19 vaccination and SARS-CoV-2 infection. The incidence of GBS is compared with mRNA-based and NVV vaccines, as well as the incidence observed after SARS-CoV-2 infection. Additionally, the risk of GBS in unvaccinated individuals who have experienced COVID-19 is compared to vaccinated individuals and assess the degree of underreporting by contrasting actively identified cases with those reported to the regional pharmacovigilance system. By addressing both methodological and regulatory aspects, this study contributes to current challenges and applications in pharmacoepidemiology and pharmacovigilance.

## 2. Results

### 2.1. Baseline Characteristics of Patients

**Figure 1:**
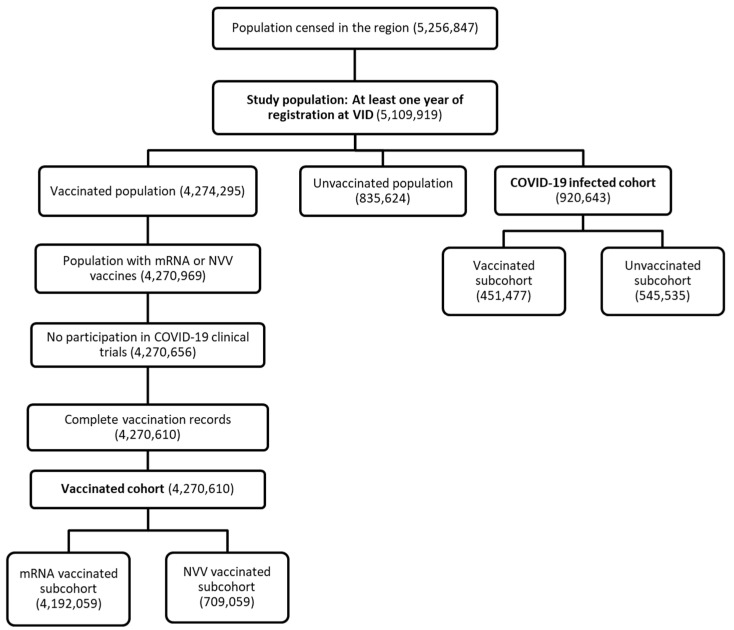
Study population flowchart and cohort selection criteria. Flow diagram of individuals registered in the Valencian Integrated Health System Database (VID) between January 2018 and March 2022. Exclusion criteria included <1 year of prior registration and vaccination outside study scope. Final cohorts comprised individuals receiving at least one COVID-19 vaccine dose (mRNA-based or NVV) and individuals with laboratory-confirmed SARS-CoV-2 infection.

Figure 1 presents the flowchart of the study population selection process and cohorts, while Table 1 details their demographic characteristics. Out of the initial population of 5,256,847 individuals registered in the Valencia region, 146,928 were excluded due to having less than one year of continuous registration in the database, ensuring adequate baseline information for exposure and outcome assessment. Additionally, 3685 individuals were excluded from the vaccinated cohort for either receiving vaccines not included in the study scope or for participating in clinical trials prior to the start of the vaccination campaign. After these exclusions, a total of 4,270,610 individuals were included in the vaccination cohort. In the COVID-19 infected cohort, 920,643 had experienced at least one COVID-19 infection by the end of the study period (Figure 1).

Table S2 categorizes the vaccines administered by dose and type. A total of 10,375,488 COVID-19 vaccine doses were recorded during the study period, with 88.92% being mRNA-based vaccines and 11.08% NVV vaccines. Among the vaccinated population, 3,839,547 (89.91%) received a second dose, and 2,265,251 (53.04%) received a third (booster) dose. The vaccine type distribution was consistent between the first and second doses, with almost all third doses being mRNA-based vaccines (Table S2).

Regarding demographic characteristics, the sex distribution was similar in both mRNA and NVV subcohorts, as well as in the general vaccinated population. However, differences were observed by age group: individuals aged 18 to 64 were more frequent in the NVV vaccinated cohort, while almost no NVV vaccines were administered to individuals aged less than 18 years old (Table 1).

Among the 920,643 individuals included in the SARS-CoV-2 infected cohort, 451,477 had been vaccinated before the infection and 545,535 were not (Figure 1). Only 33,162 patients required hospitalization. The sex distribution in COVID-19 cohort was similar to sex distribution in general population. However, only 12.57% of COVID-19 episodes occurred in individuals aged over 65 years old, reflecting a younger age distribution among those infected (Table 1). Additionally, 887,479 (96.4%) individuals experienced a single COVID-19 infection during the study period. A very small proportion (0.07% or 642 individuals) had three or more documented COVID-19 infections (Figure S1).

### 2.2. Vaccinated Cohort and GBS Incidence

**Figure 2:**
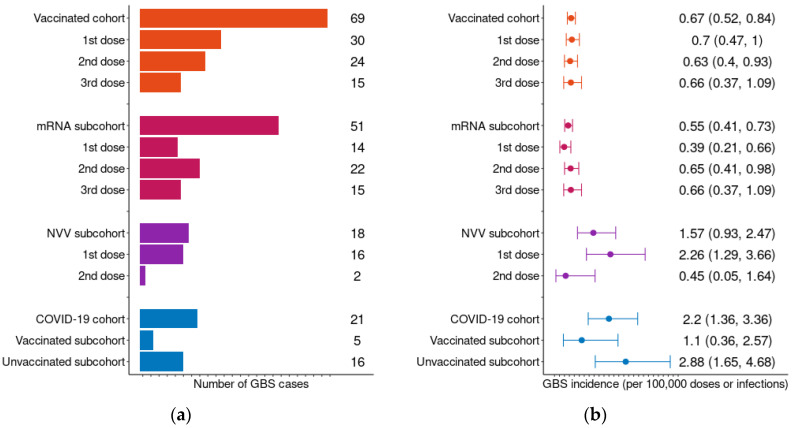
Guillain–Barré Syndrome cases and incidence within 42 days of COVID-19 vaccination or infection. Incidence expressed per 100,000 vaccine doses administered (vaccinated cohort) or per 100,000 laboratory-confirmed infections (infection cohort). Panel (**a**) shows number of GBS cases and Panel (**b**) overall incidence by exposure group and dose-specific incidence stratified by vaccine platform (mRNA-based and NVV). Only cases occurring within the predefined 42-day risk window were included.

During the study period, a total of 1136 incident GBS cases were identified across our study population from 1 January 2018 to 22 March 2022. The distribution of GBS cases and incidence, along with their 95% confidence intervals (CIs), across the different study cohorts are presented in Figure 2. In addition, the number of doses administered by dose and vaccine platform are detailed in Table 2.

Within the vaccinated cohort, 69 GBS cases occurred within the predefined 42-day risk window following vaccination, corresponding to an overall incidence of 0.67 cases per 100,000 vaccinated individuals (95% CI: 0.52–0.84). Of these, 51 cases occurred following an mRNA-based vaccine, corresponding to an incidence of 0.55 cases per 100,000 mRNA doses (95% CI: 0.41–0.73). In contrast, 18 cases were reported following an NVV vaccine, yielding a higher incidence of 1.57 cases per 100,000 NVV doses (95% CI: 0.93–2.47). This pattern persisted when the data were further stratified by sex (Table S3), with a slightly higher incidence among women than men, although confidence intervals overlapped (0.70 [0.50–0.97] vs. 0.62 [0.42–0.88] in vaccinated; 0.59 [0.39–0.85] vs. 0.52 [0.33–0.77] in mRNA vaccinated; and 1.61 [0.77–2.96] vs. 1.52 [0.65–2.99] in NVV vaccinated). Similarly, when categorized by age, the incidence of GBS was consistently higher among NVV vaccinations in almost all age groups (Table S4).

Within the mRNA vaccinated subcohort, GBS cases were more commonly observed after the administration of the second and the third dose, regardless of the vaccine type received in previous doses. When mRNA subcohort was stratified by sex and age, GBS incidence was similar, remaining higher after the second and third doses. In contrast, GBS cases were more frequent after the first dose in the NVV vaccinated subcohort. Notably, the highest incidence of GBS within the vaccinated cohort was observed following the first dose of an NVV vaccine, with an incidence of 2.26 cases per 100,000 NVV doses administered (95% CI: 1.29–3.66) (Figure 2). This represents the highest incidence among all vaccine doses when the mRNA and NVV vaccinated subcohorts are stratified by sex and age (Tables S3 and S4). Interpretation of dose-specific incidence should take into account that the primary vaccination schedule differed between vaccine brands, with a two-dose primary series for mRNA-based vaccines and ChAdOx1-S, and a single-dose primary schedule for Ad26.COV2.S.

### 2.3. COVID-19 Cohort and GBS Incidence

In the COVID-19 infected cohort, 21 GBS cases were identified within the 42-day risk window following the infection, resulting in an incidence of 2.20 cases per 100,000 COVID-19 infections (95% CI: 1.36–3.36). Of these cases, 5 individuals had been vaccinated prior to contracting COVID-19 and subsequently developing GBS, while 16 individuals had not been vaccinated before their infection. Consequently, the incidence of GBS was higher among unvaccinated individuals (2.88 cases per 100,000 infections; 95% CI: 1.65–4.68) compared to those who had been vaccinated (1.10 cases per 100,000 infections; 95% CI: 0.36–2.57) (Figure 2). Furthermore, all GBS cases occurred after the first COVID-19 infections, despite some patients experiencing multiple infections. When stratified by sex and age, the incidence of GBS was also higher among unvaccinated individuals with COVID-19 (Tables S3 and S4). Moreover, GBS incidence increased by age in unvaccinated patients with COVID-19 (0.73 [0.02–4.08] in <18 years; 2.61 [1.19–4.95] in 18–64 years; and 8.18 [3.00–17.80] in +65 years). The width of the confidence intervals reflects the small number of cases in these subgroups.

GBS incidence by COVID-19 severity (hospitalized patients vs. non-hospitalized) is shown in Table S5. Among hospitalized patients, 12 GBS cases were identified within the 42-day risk window following COVID-19 infection (incidence: 35.85 per 100,000 infections; 95 CI%: 18.51–68.94), while 9 GBS cases were observed among non-hospitalized patients (incidence: 0.98 per 100,000 infections; 95% CI: 0.45–1.86). When stratified by sex, incidence was higher among females in both groups, although confidence intervals overlapped (hospitalized: 31.68 [18.51–68.94] vs. 41.21 [15.13–89.68]; non-hospitalized: 0.93 [0.25–2.37] vs. 1.02 [0.33–2.39]). Among COVID-19 hospitalized patients, GBS incidence was highest in individuals aged 18–64 (55.26 per 100,000 infections; 95% CI: 23.86–108.86), while among non-hospitalized COVID-19 patients, the highest incidence was observed in those aged over 65 years (3.17 per 100,000 infections; 95% CI: 0.65–9.26).

The distribution of time to GBS onset within the 42-day risk window is shown in Figure S2. Among vaccinated individuals (n = 69), the majority of cases occurred within the first weeks following vaccination. The median time to onset was 21 days (interquartile range [IQR]: 12–29 days). Among individuals with laboratory-confirmed SARS-CoV-2 infection (n = 21), the median time to onset was 18 days (IQR: 4–29 days). No distinct late clustering pattern was observed within the predefined risk window.

### 2.4. GBS Severity

Hospitalization due to GBS was less frequent in the vaccinated cohort than in the infected cohort (50.72% vs. 66.67%). Within the vaccinated cohort, NVV vaccinated patients had a higher hospitalization rate than mRNA vaccinated patients (66.67% vs. 45.09%). In the infected cohort, hospitalization occurred predominantly among unvaccinated patients (68.75%), whereas only one vaccinated patient required admission (20.0%) (Table 3).

Intensive care unit (ICU) admission was slightly more frequent in the infected cohort (12.5%) and ocurred only among unvaccinated infected patients.

Median length of stay was longer in the infected cohort (15.0 [6.5–22.0] days) than in the vaccinated cohort (9.0 [4.2–19.0] days), with the longest stays observed in unvaccinated infected patients (21.0 [6.0–22.0] days).

### 2.5. Relative Risk

**Figure 3:**
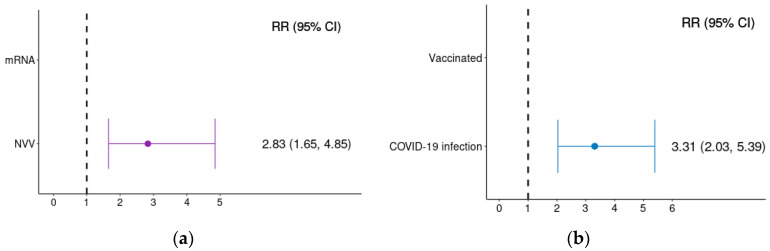
Unadjusted risk of developing GBS in the 1–42 days after exposure. Panel (**a**) shows RR by vaccine type and panel (**b**) shows RR by exposure type (COVID-19 vaccination or infection). RRs were calculated using incidence per 100,000 doses or infections, with 95% confidence intervals. RRs are represented by dots and 95% CI are represented by solid lines.

Unadjusted Relative Risk (RR) of developing GBS within 42 days following exposure, along with their 95% CIs, are presented in Figure 3. The risk of GBS was significantly higher following COVID-19 infection compared with COVID-19 vaccination (RR: 3.31; 95% CI: 2.03–5.39).

Within the vaccinated cohort, adenoviral vector vaccines were associated with a higher risk of GBS compared with mRNA-based vaccines (RR: 2.81; 95% CI: 1.65–4.85).

### 2.6. GBS Cases Reported to the PAC-VR as AEFI

During the study period, the Pharmacovigilance Autonomic Center of the Valencia Region (PAC-VR) received 23 reports of suspected GBS cases following COVID-19 vaccination. Of these, four reports were excluded (two due to latency periods exceeding 42 days and two due to missing onset dates). Consequently, 19 GBS cases were reported compared to the 69 identified through active surveillance in the VID, corresponding to a reporting rate of 27.54% for severe adverse events (Figure S3). Reporting proportions differed markedly by vaccine platform, with 61.11% of adenoviral vector-related cases reported, compared with 15.69% of mRNA-related cases (Figure S3). Given the limited number of reported cases, further stratification by age and sex was not performed, as it would have resulted in very small cell counts and unstable estimates.

Upon notification, the recovery status was conducted for the 19 suspected cases of GBS reported to the PAC-VR. Of these cases, 16 required hospitalization before the notification date (Figure S4). At the time of notification, 10 patients were already in the process of recovery, with one having fully recovered without any residual effects. Additionally, two individuals had recovered but still experienced residual symptoms, while six had not shown signs of improvement.

## 3. Discussion

In this large population-based cohort study, we evaluated the incidence and relative risk of Guillain–Barré Syndrome (GBS) following COVID-19 vaccination and SARS-CoV-2 infection using routinely collected healthcare data in the Valencia region of Spain. Our findings indicate that GBS following COVID-19 vaccination was rare and occurred less frequently than GBS following a SARS-CoV-2 infection. In addition, we observed a higher incidence and relative risk of GBS after NVV vaccines compared with mRNA-based vaccines, which is consistent with previous studies [22]. This observational study reinforces the importance of contextualizing vaccine safety signals against the background risk associated with the infection itself. Although COVID-19 vaccination has been temporally associated with rare neurological adverse events, our results support the evidence that the overall risk of GBS is markedly greater following a COVID-19 infection, particularly among unvaccinated individuals.

The crude incidence of GBS following COVID-19 vaccination in our study (0.67 per 100,000 doses) was higher than that reported in some Asian populations, such as the Korean surveillance study by Jongmok Ha et al. (0.14 per 100,00 doses) [23]. Although our incidences are higher, this article also highlights the increased risk associated with NVV vaccines, with an incidence of 0.45 per 100,000 doses compared to mRNA-based vaccines at 0.08 per 100,000 doses. In our study, the values were 1.57 and 0.55 per 100,000 doses, respectively. Differences in incidence estimates across studies may reflect variations in population characteristics, healthcare-seeking behavior, and the completeness of outcome ascertainment in different data sources.

The temporal distribution of GBS onset following both vaccination and infection showed that most cases occurred within the first weeks after exposure, consistent with the expected time frame of immune-mediated neurological complications [24]. However, given the limited number of events, this descriptive finding should be interpreted cautiously.

GBS also appears to occur primarily after an initial COVID-19 infection, with no cases identified in subsequent reinfections in our sample. This finding aligns with the study by Jongmok Ha et al., which reported that GBS following vaccination was more frequent after the first dose, while subsequent doses appeared to provide some protective effect against the development of GBS [23]. However, given the rarity of events and overlapping confidence intervals across dose categories in our study, we avoid inferring a true protective dose effect. When examining dose-specific incidence patterns, the interpretation requires caution. Although the overall incidence following NVV vaccines was higher than that observed for mRNA-based vaccines, dose-stratified analyses showed variability across successive doses. In particular, the apparent reduction in incidence after the second NVV dose must be interpreted in light of the very small event numbers and wide confidence intervals. After stratification by sex, no cases were observed among men and only two cases among women after the second NVV dose, resulting in unstable incidence estimates. In contrast, mRNA vaccines were administered in substantially larger numbers across all doses, yielding more stable incidence patterns. These findings likely reflect a combination of statistical imprecision and programmatic factors rather than a true biological protective effect. NVV vaccines were predominantly used for first doses during the early phases of the vaccination campaign, with a later shift towards mRNA-based boosters. As a result, individuals receiving a second NVV dose constituted a smaller and potentially selected subgroup. A “depletion-of-susceptibles” phenomenon may also contribute, whereby individuals predisposed to immune-mediated adverse reactions manifest symptoms after the first exposure and are unlikely to receive subsequent doses. Similar patterns have been described in the context of other vaccine-associated rare adverse events (e.g., influenza vaccines and GBS), where clustering tends to occur after initial immune stimulation rather than repeated exposure [25].

Our results also suggest that vaccination may help prevent post-COVID-19 GBS, as the incidence was higher among unvaccinated individuals compared to those who were vaccinated before infection. However, the possibility of survivor bias should be considered, as individuals predisposed to developing GBS may have already done so following vaccination, leaving a less susceptible population for subsequent analyses.

Although age- and sex-stratified analyses were performed, the small number of events limited statistical precision and precluded adjusted modeling. These findings should therefore be interpreted with caution.

The reporting percentages for post-vaccination adverse event to the Pharmacovigilance Autonomic Center of the Valencia Region (PAC-VR) were very low, with only 27.54% of the cases in the VID being reported as suspected adverse events, translating into a 73.46% underreporting rate. This is particularly concerning given that GBS is a serious adverse event and was relatively unknown as a risk for these vaccines at that time. In comparison, other studies that examined the COVID-19 vaccination notification rates had found significantly higher reporting percentages for moderate to severe AEFIs, with some reporting 50.5% of moderate to severe AEFIs after the first dose [26]. The difference in reporting percentages between NVV and mRNA-based vaccines is particularly striking. This could be attributed to greater attention being paid to NVV vaccines (it would be understandable given that these vaccines had raised greater social alarm due to cases of thrombosis with thrombocytopenia in uncommon anatomical sites, and GBS itself was described in their technical product information [18,19]. E.R. Miller et al. also found a different reporting ratio of GBS following the administration of different vaccines, with ratios ranging from 12% to 64% [27].

Pharmacovigilance is primarily based on the study of spontaneous reporting and the clinical and pharmacological analysis of a drug’s responsibility in the occurrence of individual cases of adverse events. Through this process, it provides unparalleled benefits in identifying safety signals, even for rare events or those associated with infrequently used medications. However, there is a recognized tendency for underreporting events in pharmacovigilance. Pharmacoepidemiology, with its real-world data population-focused approach and the potential use of a comparison group, allows for a quantification of risks that is impossible to achieve based solely on spontaneous reporting data. It also enables confirmed risks, to specify the importance of these risks and their impact on the health of populations and an assessment of the drug’s benefit [28]. However, the fact that the cases from the active search in VID are not manually validated may be influencing both the notification rates (there may be cases detected as such by physicians that do not actually have sufficient evidence to be considered GBS and therefore have not been reported) and the difference in notification percentages between both types of vaccines, if these false positives are distributed unevenly between the two groups. Further case validation is needed to confirm our findings.

This study has several strengths. It was conducted using a large population-based healthcare database covering more than 95% of the regional population (approx. 5 million inhabitants), with mandatory registration of COVID-19 vaccinations across public and private settings. Vaccine brand, date, and dose number are systematically recorded, minimizing exposure misclassification. Laboratory-confirmed SARS-CoV-2 infections and a predefined 42-day risk window ensured consistent exposure definitions. In addition, individuals with prior GBS diagnoses were excluded and at least one year of prior registration was required to strengthen identification of incident cases.

However, several limitations should be considered. Outcome identification relied exclusively on ICD diagnostic codes without individual clinical validation. Although coding practices are standardized and unlikely to differ systematically between exposure groups, some non-differential misclassification cannot be excluded.

Because only laboratory-confirmed SARS-CoV-2 infections were included, undiagnosed or untested infections may not have been captured. Therefore, the reported estimates reflect the risk among confirmed infections and may not be directly generalizable to all SARS-CoV-2 infections.

Finally, due to the rarity of GBS and the limited number of events, adjusted multivariable analyses were not feasible. Several exposure subgroups contained very few or no cases, which would have resulted in unstable or unreliable regression estimates. Although stratified incidence estimates by age and sex are presented, residual confounding cannot be excluded. The use of a uniform 42-day risk window across exposure groups reduces temporal variability but does not eliminate the possibility of unmeasured confounding. Although the database allowed estimation of pre-pandemic GBS incidence, formal attributable risk comparisons with historical background rates were beyond the primary objective of this study and warrant further investigation. Despite its limitations, this study underscores that spontaneous reporting systems alone are inadequate to ensure that severe adverse events such as GBS following COVID-19 vaccination are adequately reported. Pharmacoepidemiologic studies like the one presented, that leverage robust population-based real-world data, can improve awareness and help mitigate underreporting issues.

## 4. Materials and Methods

### 4.1. Study Design, Cohort and Population

This population-based retrospective cohort study used real-world data from the Valencian Health System (VID) [21], which collects and integrates health information for the Valencia region (Spain). The study period covered from 1 January 2018 to 22 March 2022, allowing for a broad analysis of the incidence of GBS in relation to both COVID-19 vaccinations and SARS-CoV-2 infection. The study period began in January 2018 to ensure at least one year of prior registration for all individuals, enabling accurate identification of incident GBS cases and reducing the likelihood of including prevalent cases. The pre-pandemic period was not intended for formal background incidence comparison but rather to strengthen case ascertainment and cohort definition.

COVID-19 vaccines were categorized into two platforms: mRNA-based vaccines and non-virus-vectored (NVV) vaccines. mRNA-based vaccines included BNT162b2 (Pfizer Inc., New York, NY, USA/BioNTech SE, Mainz, Germany) and mRNA-1273 (Moderna, Cambridge, MA, USA). For the purposes of this study, NVV vaccines correspond to adenoviral vector-based COVID-19 vaccines, including ChAdOx1-S (AstraZeneca, Cambridge, UK) and Ad26.COV2.S (Janssen Pharmaceuticals, Beerse, Belgium). These two platforms were analyzed separately due to previously reported differences in safety signals related to immune-mediated neurological events. During the study period, the standard primary vaccination schedule consisted of two doses for mRNA-based vaccines (BNT162b2 and mRNA-1273) and ChAdOx1-S, and a single dose for Ad26.COV2.S, in accordance with national vaccination guidelines. Subsequent booster doses were predominantly mRNA-based vaccines.

Two different study cohorts were considered: individuals receiving at least one COVID-19 vaccine dose (mRNA-based or NVV) and (2) individuals with laboratory-confirmed SARS-CoV-2 infection, regardless of vaccination status. The vaccination cohort was subdivided into two overlapping subcohorts: those who received at least one dose of an mRNA-based vaccine and those who received at least one dose of an NVV vaccine. The COVID-19 infected cohort was also stratified into two subcohorts: those individuals vaccinated before the infection and those who either remained unvaccinated or were vaccinated after the infection.

Eligibility required at least one year of continuous registration in the VID prior to cohort entry. Individuals who received vaccines outside the study scope or participated in COVID-19 clinical trials before the public vaccination campaign were excluded from the vaccinated cohort.

The start of follow-up differed between the two cohorts. For the vaccinated cohort, follow-up began on the latest of the following dates: 27 December 2020 (the start of COVID-19 vaccination campaign in the Valencia region), the individual’s registration date in the VID and the individual’s birth date. For the COVID-19 infected cohort, follow-up began on the latest of these dates: 9 February 2020 (date of the first COVID-19 diagnosis recorded in the VID), the individual’s inclusion date in the VID or the individual’s birth date. In both cohorts, the end of follow-up was defined as the earliest of the following dates: 22 March 2022, the individual’s deregistration date in the VID or the individual’s death date.

Self-controlled study designs were not applied due to the rarity of Guillain–Barré syndrome and the limited number of cases, which would have compromised statistical power and precision.

### 4.2. Data Source

In Valencia (population: ~5 million, representing ~10.7% of the overall Spanish population), medical and sociodemographic information is obtained from the population-based electronic information systems of the Valencia Health Agency (VHA) and the regional Government of Valencia. These are linked to the VID [21], which covers 97% of the population of the region and provides exhaustive longitudinal data. This includes sociodemographic and administrative information (such as sex, age and nationality), clinical data (such as diagnoses, procedures, diagnostic tests and imaging), pharmaceutical records (prescription and dispensation) and healthcare utilization data from hospital care, emergency departments, specialized care (including mental and obstetrics care), primary care and other public health services such as vaccinations. This extensive data can be linked at the individual level, facilitating real-world data research [29,30,31,32].

### 4.3. Outcome

GBS cases were identified using data from general practitioners, emergency departments, hospitalizations, and outpatient records. GBS cases were identified by the following codes: 357.0 (ICD-9) and G61.0 (ICD-10) codes. Potential GBS cases with additional diagnoses of Chronic Inflammatory Demyelinating Polyneuropathy (CIDP) were excluded (ICD-codes detailed in Table S1). This rigorous exclusion process aimed to minimize the inclusion of unrelated polyneuropathies that could confound the results.

The risk window for GBS occurrence following vaccination or infection was defined as 1–42 days, in line with Brighton Collaboration recommendations [2].

### 4.4. Exposure

Vaccine exposure and COVID-19 diagnoses were extracted from the VID [21]. Vaccination records were extracted from the Vaccine Information System (VIS), which provides detailed, real-time information on all vaccinations administered. COVID-19 infections were identified through laboratory-confirmed polymerase chain reaction (PCR) tests or antigen tests, using data from the regional Microbiological Surveillance Network (RedMIVA). Two positive COVID-19 results within 90 days were considered the same episode, to avoid overcounting reinfections during short time periods. COVID-19 test results were linked to hospital records to determine which cases required hospitalization and which did not. A hospitalization was considered associated with a positive COVID-19 result if the diagnostic test was performed during admission or within the previous 15 days. Exposure data were linked to GBS cases at the individual level using the pseudo-anonymized personal identification number (SIP). This secure identifier allowed for precise linkage of medical and exposure data while preserving patient privacy in compliance with data protection regulations.

### 4.5. Statistical Analysis

Demographic characteristics (sex and age) of both study cohorts were described using frequencies and proportions. The number of doses administered was categorized by vaccine type, differentiating between mRNA-based vaccines and NVV vaccines.

GBS cases were analyzed within a 42-day risk window following exposure for both vaccinated and COVID-19 infected cohorts. For the vaccinated cohort, GBS cases were counted per dose, stratifying by vaccine type (mRNA or NVV), as well as by dose and vaccine type. In the COVID-19 infected cohort, GBS cases were analyzed also by their vaccination status (vaccinated prior to the infection or unvaccinated at the time of the infection). Cases were also examined by infection severity (hospitalized vs. non-hospitalized infections). Additionally, cumulative incidence was calculated per 100,000 doses administered or tests conducted in each subcohort (number of cases divided by the corresponding exposed population). These calculations were all repeated stratifying by sex and age group to account for potential demographic variations. Time from exposure to GBS was described, and the median and the QR were calculated for both exposure types (COVID-19 vaccinations and infection).

GBS severity was described in both cohorts. The frequency and proportion of cases requiring hospitalization or ICU admission were calculated, and length of hospital stay was described for admitted patients. Hospitalized GBS cases included all cases diagnosed during a hospital admission, as well as cases diagnosed in an outpatient setting that were followed by a GBS related hospitalization within 30 days of the initial diagnosis.

Unadjusted RR of experiencing a GBS case following NVV vaccination compared to mRNA vaccination was calculated. Additionally, the risk of experiencing GBS in the COVID-19 infected cohort compared to the vaccinated cohort was calculated. All RR were calculated with their respective 95% CI. 

To assess under-reporting to pharmacovigilance systems, the study compared GBS cases identified in the VID with individual safety reports submitted at the Pharmacovigilance Autonomic Center of the Valencia Region (PAC-VR). Only cases with onset within 42-day risk window were included to ensure consistency with study’s predefined post-vaccination case identification criteria. Cases with a latency period exceeding 42 days were excluded to avoid misclassification. All analyses were conducted on anonymized data using R software (Foundation for Statistical Computing, Vienna, Austria) version 4.3.2.

## 5. Conclusions

In this large population-based study, Guillain–Barré syndrome following COVID-19 vaccination was a rare event. The incidence was higher after adenoviral vector vaccines than after mRNA-based vaccines, but substantially lower compared to the incidence following a SARS-CoV-2 infection, particularly among unvaccinated individuals.

These findings reinforce the favorable benefit–risk profile of COVID-19 vaccines and highlight the importance of contextualizing vaccine safety signals against infection-related risks. Moreover, the substantial underreporting of GBS cases to pharmacovigilance systems underscores the need to strengthen adverse event reporting and to integrate active surveillance using real-world data with spontaneous reporting systems.

Further studies incorporating standardized clinical case validation are warranted to refine incidence estimates and to support regulatory decision-making for rare but serious adverse events.

## Figures and Tables

**Table 1:** Demographic characteristics of the study cohorts. The number of subjects, stratified by sex and age, at the end of follow-up of the general population registered in VID, the vaccinated cohort (overall and by vaccine platform), and the SARS-CoV-2 infected cohort are shown. Percentages have been calculated within each cohort.

Population	Total	Sex		Age at the End of Follow-Up		
		Males	Females	<18 Years Old	18–64 Years Old	+65 Years Old
General population	5,109,919	2,510,709 (49.13%)	2,599,210 (50.87%)	820,162 (16.05%)	3,119,006 (61.04%)	1,170,751 (22.91%)
Vaccinated cohort	4,270,610	2,077,250 (48.64%)	2,193,360 (51.36%)	505,191 (11.83%)	2,780,394 (65.11%)	985,025 (23.07%)
mRNA vaccinated	4,192,059	2,038,565 (48.63%)	2,153,494 (51.37%)	505,175 (12.05%)	2,707,060 (64.58%)	979,824 (23.37%)
NVV vaccinated	709,564	332,979 (46.93%)	376,585 (53.07%)	89 (0.01%)	622,786 (87.77%)	86,689 (12.22%)
COVID-19 infection cohort	920,643	436,356 (47.40%)	484,287 (52.60%)	175,386 (19.05%)	629,491 (68.38%)	115,766 (12.57%)
Vaccinated	451,477	206,249 (45.68%)	245,228 (54.32%)	52,311 (11.59%)	348,204 (77.13%)	50,962 (11.29%)
Unvaccinated	545,535	263,672 (48.33%)	281,863 (51.67%)	126,068 (23.11%)	341,947 (62.68%)	77,520 (14.21%)
Hospitalized	33,162	18,745 (56.53%)	14,417 (43.47%)	490 (1.48%)	13,816 (41.66%)	18,856 (56.86%)
Non-hospitalized	888,859	418,283 (47.06%)	470,576 (52.94%)	174,928 (19.68%)	616,446 (69.35%)	97,485 (10.97%)

**Table 2:** Guillain–Barré syndrome cases and incidence within 1–42 days after COVID-19 vaccination, by vaccine platform and dose number. Incidence is expressed per 100,00 vaccine doses, with 95% confidence intervals.

Vaccine Platform	Dose	GBS Cases (n)	Doses Administered (n)	Incidence per 100,000 Doses	95% CI
mRNA	Dose 1	14	3,561,591	0.39	(0.21–0.66)
mRNA	Dose 2	22	3,398,817	0.65	(0.41–0.98)
mRNA	Dose 3	15	2,265,251	0.66	(0.37–1.09)
NVV	Dose 1	16	709,019	2.26	(1.29–3.66)
NVV	Dose 2	2	440,730	0.45	(0.05–1.64)
Overall vaccinated	All doses	69	10,375,408	0.67	(0.52–0.84)

**Table 3:** Description of GBS hospitalizations within 42 days after exposure (COVID-19 vaccination or infection), overall and by study subcohorts.

	Vaccinated Cohort			COVID-19 Infected Cohort		
	Total, N = 69	RNA Subcohort, N = 51	NVV Subcohort, N = 18	Total, N = 21	Vaccinated Subcohort, N = 5	Not Vaccinated Subcohort, N = 16
Hospitalizations, n (%)	35 (50.72%)	23 (45.09%)	12 (66.67%)	12 (66.67%)	1 (20.00%)	11 (68.75%)
ICU admission, n (%)	5 (7.25%)	4 (7.84%)	1 (5.56%)	2 (9.52%)	0 (0.00%)	2 (12.50%)
Length of stay, Median (IQR)	9.0 (4.2–19.0)	8.5 (4.0–15.5)	10.5 (7.0–21.8)	15.0 (6.5–22.0)	9.0 (9.0–9.0)	21.0 (6.0–22.0)

## Data Availability

The original contributions presented in this study are included in the article/Supplementary Material. Further inquiries can be directed to the corresponding authors.

## Supplementary Materials

The following supporting information can be downloaded at: https://www.mdpi.com/article/10.3390/ph19030477/s1, Figure S1: Distribution of reported COVID-19 infections during our study period (from 9 February 2020 to 22 March 2022); Figure S2: Time from exposure to Guillain–Barré syndrome onset within the 42-day risk window; Figure S3: GBS cases in vaccinated cohort and subcohorts in the 42-day risk window by datasource (PAC-VR and data obtained from VID); Figure S4: Need of hospitalization in reported cases from PAC-VR of suspected GBS post-vaccination within the 42-day risk window at the time of notification; Table S1: ICD-9 and ICD-10 codes for inclusion and exclusion of GBS cases; Table S2: Number ofCOVID-19 vaccine doses administered during our study period grouped by vaccine platform and dose number; Table S3: GBS cases and incidence within 42 days after exposure (COVID-19 vaccination or infection), stratified by sex; Table S4: GBS cases and incidence within 42 days after exposure (COVID-19 vaccination or infection), stratified by age group, COVID-19 vaccination and infection; Table S5: GBS cases and incidence within 42 days after COVID-19 infection, stratified by COVID-19 hospitalization.

## Abbreviations

The following abbreviations are used in this manuscript:

AEFI	Adverse Event Following Immunization
AESI	Adverse Event of Special Interest
CI	Confidence Interval
GBS	Guillain–Barré syndrome
ICU	Intensive care unit
IQR	Interquartile range
NVV	Non-virus-vectored (adenoviral vector–based) COVID-19 vaccines
PAC-VR	Pharmacovigilance Autonomic Center of the Valencia Region
PCR	Polymerase Chain Reaction
RR	Relative Risk
VID	Integrated Database of the Valencian Health System
VIS	Vaccine Information System
