# Supplementary material for: Incidence of Guillain–Barré Syndrome Following COVID-19 Vaccination and SARS-CoV-2 Infection: A Population-Based Cohort Study Using the Valencia Health System Integrated Database (Spain)
Source: Pharmaceuticals (Basel). 2026 Mar 14;19(3):477. doi: 10.3390/ph19030477 (PMC13029112; doi:10.3390/ph19030477)
Supplement: Supplementary file 1 [file pharmaceuticals-19-00477-s001.zip › pharmaceuticals-4142796-supplementary.pdf]

Incidence of Guillain–Barré Syndrome Following Covid-19 Vaccination and SARS-CoV-2 Infection: A Population-Based Cohort Study Using the Valencia Health System Integrated Database (Spain)

Supplementary Tables and Figures

Table S1. ICD-9 and ICD-10 codes used for the inclusion and exclusion of GBS cases.

ICD-9	ICD-10	Description	Inclusion/Exclusion
357.0	G61.0	Guillain-Barré syndrome	Inclusion
357.8	G61.8	Other inflammatory and toxic neuropathy/ Other inflammatory polyneuropathies	Exclusion
357.9	G61.9	Inflammatory polyneuropathy, unspecified	Exclusion
-	G62.8	Other specified polyneuropathies	Exclusion
-	G62.9	Polyneuropathy, unspecified	Exclusion

Table S2. Number of COVID-19 vaccine doses administered during our study period(*) grouped by vaccine platform and dose number.

Vaccine doses	Both platforms	mRNA vaccines, N (%)	NVV vaccines, N (%)
Dose 1	4,270,610	3,561,591 (83.4%)	709,019 (16.6%)
Dose 2	3,839,547	3,398,817 (88.5%)	440,730 (11.5%)
Dose 3	2,265,251	2,265,251 (100%)	0 (0%)
Total doses administered	10,375,408	9,225,659 (88.9%)	1,149,749 (11.1%)

(*) Only the study period which vaccines were available was considered (from 27 December 2020 to 22 March 2022).

Table S3. GBS cases and incidence within 42 days after exposure (Covid-19 vaccination or infection), stratified by sex. Incidence rates are presented separately for vaccination (per 100,000 doses administered) and SARS-CoV-2 infection (per 100,000 laboratory-confirmed infections), with 95% confidence intervals.

Cohort/subcohort	GBS cases		GBS incidence (95% CI)	
	Males	Females	Males	Females
**Vaccinated**	31	38	0.62 (0.42, 0.88)	0.70 (0.50, 0.97)
Dose 1	13	17	0.63 (0.33, 1.07)	0.78 (0.45, 1.24)
Dose 2	11	13	0.59 (0.30, 1.06)	0.65 (0.35, 1.12)
Dose 3	7	8	0.66 (0.27, 1.37)	0.66 (0.29, 1.30)
**mRNA vaccinated**	23	28	0.52 (0.33, 0.77)	0.59 (0.39, 0.85)

Dose 1	5	9	0.29 (0.09, 0.67)	0.50 (0.23, 0.94)
Dose 2	11	11	0.66 (0.33, 1.19)	0.63 (0.32, 1.13)
Dose 3	7	8	0.66 (0.27, 1.37)	0.66 (0.29, 1.30)
**NVV vaccinated**	8	10	1.52 (0.65, 2.99)	1.61 (0.77, 2.96)
Dose 1	8	8	2.40 (1.04, 4.74)	2.13 (0.92, 4.19)
Dose 2	0	2	0.00	0.81 (0.10, 2.94)
**Covid-19 infected cohort**	10	11	2.22 (1.06, 4.08)	2.18 (1.09, 3.91)
Vaccinated	1	4	0.48 (0.01, 2.69)	1.62 (0.44, 4.14)
Unvaccinated	9	7	3.36 (1.53, 6.37)	2.44 (0.98, 5.02)

Table S4. GBS cases and incidence within 42 days after exposure (COVID-19 vaccination or infection), stratified by age group. Incidence is expressed per 100,000 vaccine doses or laboratory-confirmed infections, with 95% confidence intervals.

**Cohort/subcohort**	**GBS cases**			**GBS incidence (95% CI)**		
	**< 18 years old**	**18-64 years old**	**+65 years old**	**< 18 years old**	**18-64 years old**	**+65 years old**
**Vaccinated**	5	41	23	0.62 (0.20, 1.44)	0.60 (0.43, 0.81)	0.85 (0.54, 1.28)
Dose 1	3	21	6	0.56 (0.12, 1.65)	0.75 (0.46, 1.15)	0.64 (0.24, 1.39)
Dose 2	2	12	10	0.74 (0.09, 2.69)	0.46 (0.24, 0.80)	1.06 (0.51, 1.95)
Dose 3	0	8	7	0.00	0.58 (0.25, 1.13)	0.80 (0.32, 1.66)
**mRNA vaccinated**	5	24	22	0.62 (0.20, 1.44)	0.42 (0.27, 0.62)	0.83 (0.52, 1.26)
Dose 1	3	6	5	0.56 (0.12, 1.65)	0.28 (0.10, 0.61)	0.56 (0.18, 1.31)
Dose 2	2	10	10	0.75 (0.09, 2.69)	0.45 (0.22, 0.83)	1.09 (0.52, 2.01)
Dose 3	0	8	7	0.00	0.58 (0.25, 1.13)	0.80 (0.32, 1.66)
**NVV vaccinated**	0	17	1	0.00	1.56 (0.91, 2.50)	1.69 (0.04, 9.41)
Dose 1	0	15	1	0.00	2.25 (1.26, 3.72)	2.39 (0.06, 12.84)
Dose 2	0	2	0	0.00	0.48 (0.06, 1.74)	0.00
**Covid-19 infected cohort**	1	13	7	0.53 (0.01, 2.94)	1.99 (1.06, 3.41)	6.35 (2.55, 13.08)

Vaccinated	0	4	1	0.00	1.14 (0.31, 2.92)	1.98 (0.05, 11.06)
Unvaccinated	1	9	6	0.73 (0.02, 4.08)	2.61 (1.19, 4.95)	8.18 (3.00, 17.80)

Table S5. Number of GBS cases and incidence within 42 days after a COVID-19 infection, stratified by COVID-19 hospitalization status. Incidence is expressed per 100,000 laboratory-confirmed infections, with 95% confidence intervals. Incidence is calculated overall and by sex and age group.

	**GBS cases**		**GBS incidence**	
	**Hospitalized**	**Not hospitalized**	**Hospitalized**	**Not hospitalized**
**Overall**	12	9	35.82 (18.51, 68.94)	0.98 (0.45, 1.86)
**Sex**				
Males	6	4	31.68 (11.63, 68.94)	0.93 (0.25, 2.37)
Females	6	5	41.21 (15.13, 89.68)	1.02 (0.33, 2.39)
**Age group**				
< 18 years old	0	1	0.00	0.01, 2.95
18-64 years old	8	5	55.26 (23.86, 108.86)	0.78 (0.25, 1.83)
+65 years old	4	3	21.62 (5.89, 55.35)	3.17 (0.65, 9.26)

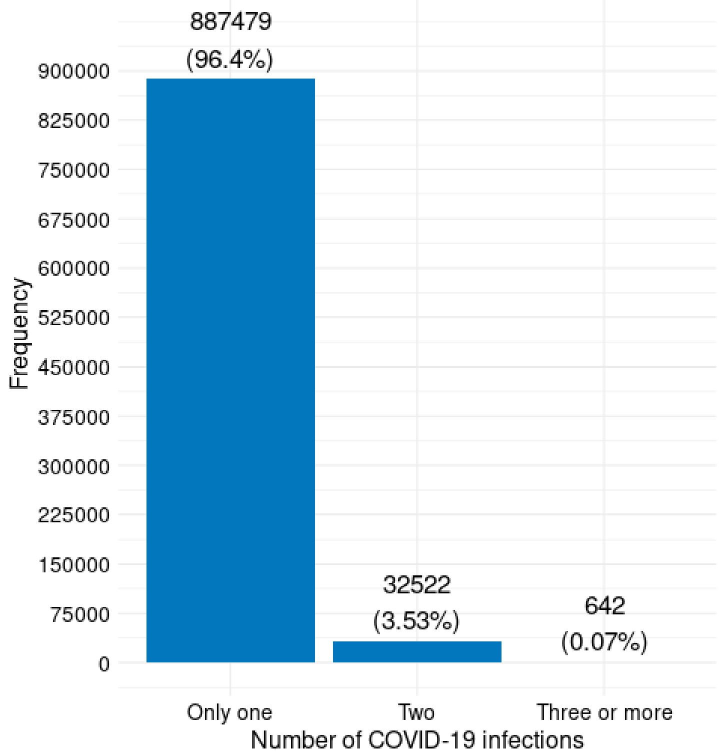

Figure S1. Distribution of reported COVID-19 infections during the study period (from 9 February 2020 to 22 March 2022).

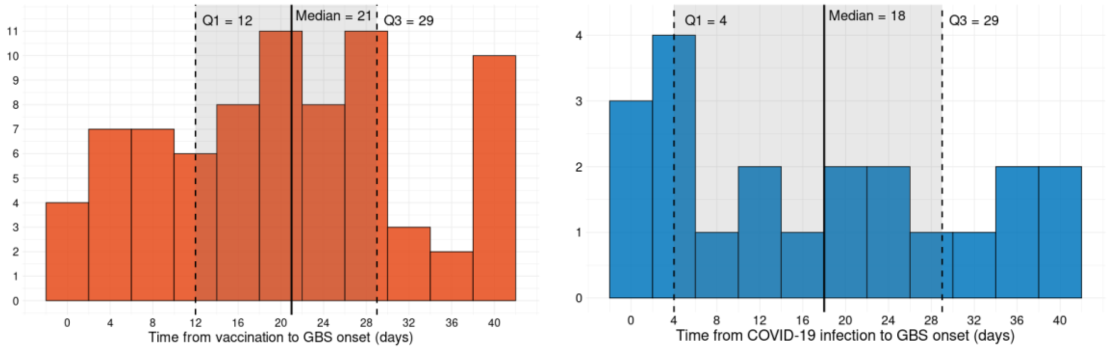

(**a**) (**b**)

Figure S2. Time from exposure to Guillain–Barré syndrome onset within the 42-day risk window. Panel (a) shows distribution of days from COVID-19 vaccination (any platform, all doses combined; n = 69 cases) to first recorded GBS diagnosis; and (b) distribution of days from laboratory-confirmed SARS-CoV-2 infection (n = 21 cases) to first recorded GBS diagnosis.

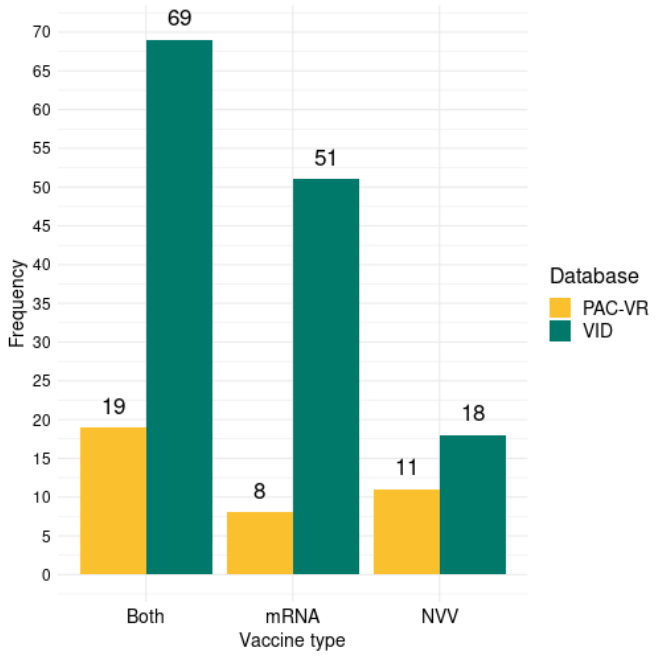

Figure S3. GBS cases in the vaccinated cohort and subcohorts in the 42-day risk window by data source. Green bars: The Valencia Health System Integrated Database (VID), yellow bars: Pharmacovigilance Autonomic Center of the Valencia Region (PAC-VR)

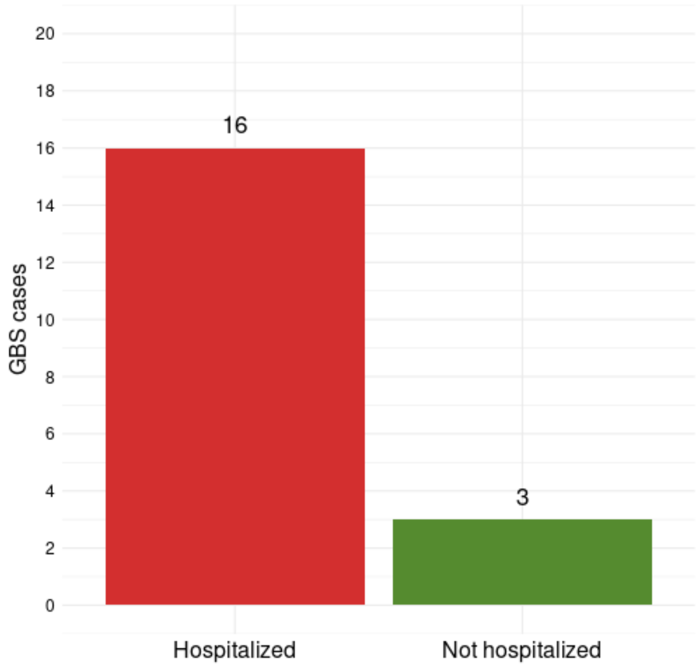

Figure S4. Hospitalization status among the cases of suspected GBS post-vaccination within the 42-day risk window notified to the Pharmacovigilance Autonomic Center of the Valencia Region (PAC-VR).
